# Correction: DNA barcodes for the fishes of the Narmada, one of India's longest rivers

**DOI:** 10.1371/journal.pone.0105490

**Published:** 2014-08-08

**Authors:** The PLOS ONE Staff

There is an error in the title. The word Fishes has an incorrectly capitalized I. The correct title should be “DNA Barcodes for the Fishes of the Narmada, One of India's Longest River.”

The correct Citation should read: Khedkar GD, Jamdade R, Naik S, David L, Haymer D (2014) DNA Barcodes for the Fishes of the Narmada, One of India’s Longest Rivers. PLoS ONE 9(7): e101460. doi:10.1371/journal.pone.0101460

Additionally, there are errors in Figure 2. Please see the corrected Figure 2 here.

**Figure 2:**
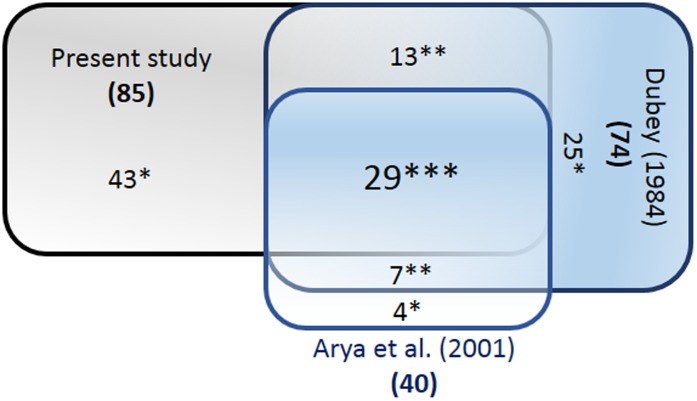
Comparison of fish species with species records from earlier studies. (*specific species to the study; **common species for two studies; ***common species for all studies).
